# Efficiency and Suitability when Exploring the Conformational Space of Phase-Transfer Catalysts

**DOI:** 10.1021/acs.jcim.2c00934

**Published:** 2022-10-22

**Authors:** Iñigo Iribarren, Cristina Trujillo

**Affiliations:** School of Chemistry, Trinity College Dublin, College Green, Dublin2, Ireland

## Abstract

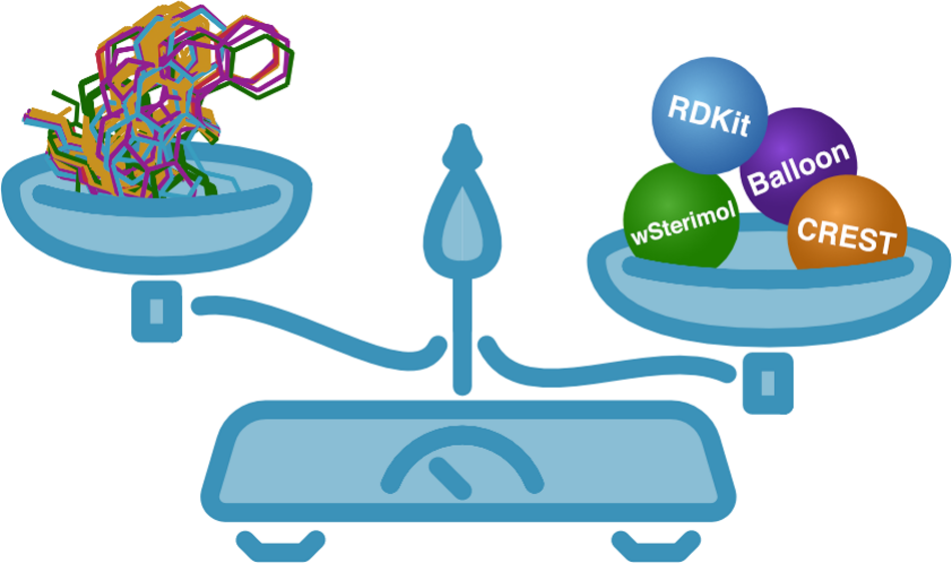

In this study, a complete exploration of the conformational space of different phase-transfer catalysts by means of computational method benchmarking is presented. For this particular research work, only the most significant and relevant conformational analysis approaches have been chosen to characterize the main Cinchona alkaloid-based phase-transfer catalysts. This particular guiding study aims to rigorously compare the performance of different conformational methods, determining the strengths of each method and providing recommendations regarding suitable and efficient choices of methods for analysis.

## Introduction

Conformational analysis plays a fundamental role in various research fields of organic chemistry. The first definition of conformation was proposed by Barton: “the conformations of a molecule (of defined constitution and configuration) are those arrangements in space of the atoms of the molecule which are not superimposable upon each other”.^1,2^

Organocatalysts are flexible compounds with rich structural dynamics. Similarly to enzymes, their function is directly linked to their structure and their activity may be regulated via their conformational dynamics. Consequently, it is crucial to investigate their full conformational space when studying such flexible molecules. The generation of conformations for small molecules is a challenging problem of continuing interest in cheminformatics and computational drug discovery. This study will present an overview of methods used to sample conformational space, focusing on those methods designed for organic molecules commonly of interest in conformational analysis.

Phase-transfer catalysts (PTCs) were introduced by Starks for the first time in 1971.^3^ They are systems that work between two liquid immiscible phases and have been widely applied in organic reactions. Particularly, asymmetric PTCs, catalysts presenting well-defined chiral motifs, have been recognized as an adaptable and capable procedure for preparing chiral functional molecules. As a result, many novel organic transformations have been achieved with high enantioselectivity.^4−8^ Among the different PTC classes developed, chiral binaphthyl and Cinchona alkaloid have been proven as the most successful examples, including the quaternary ammonium salts,^4,9−14^ and have been successfully applied to highly enantioselective conversions.^15^

**Figure 1:**
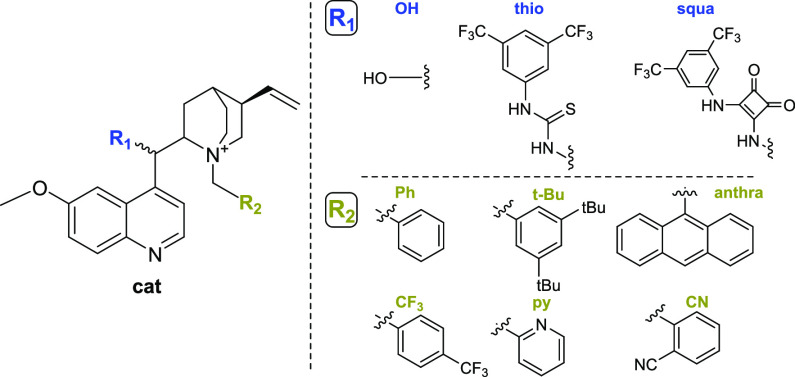
Chiral bifunctional PTCs under study.

For this particular benchmark, three different types of PTCs have been chosen to cover the most commonly used Cinchona alkaloid-based derivatives (Figure 1).

**Figure 2:**
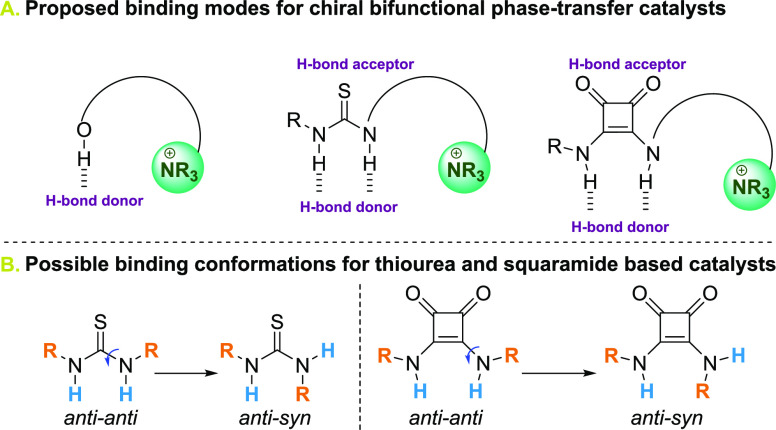
(A) Proposed binding modes for chiral bifunctional PTCs under study. (B) Two different binding conformations for thiourea and squaramide-based PTCs.

Those particular PTCs chosen for this benchmark are well-known examples within the traditional hydrogen bonding-based catalysis field and the binding mode via a bifurcated hydrogen bond has been confirmed in the literature (Figure 2A).^16^ In particular, thiourea and squaramide units have been considered as very efficient bifunctional catalysts for several important enantioselective organic transformations.^17−25^

In fact, it has been found that both scaffolds can adopt *anti–anti* and *anti–syn* conformations, and it is clear that the conformations adopted by thiourea and squaramide functional groups influence both the catalysis and binding (Figure 2B). Ureas are less frequently observed in the aforementioned *syn–anti* conformation. This is not the case, however, for thioureas, which exhibit a more varied conformational behavior.^26^ Luchini et al. performed a computational study of the different conformers that can be adopted by diarylureas and diarylthioureas, concluding that diarylureas exhibit an overwhelming preference for the *anti–anti* conformer, while the diarylthioureas show a mixture of *anti–anti* and *anti–syn* conformers, and the proportion of the *anti–anti* conformer is predicted to increase with solvent polarity/Lewis basicity.^27^ The accepted mechanism of Schreiner’s catalyst, however, features a double hydrogen bond to the substrate that only forms with the *anti–anti* conformation of its central thiourea group.^28^

Given the inherent conformational problem featured within the different thiourea/squaramide-based catalysts and the impact on the possible binding mode, an exploration of the conformational space within PTCs by means of computational method benchmarking is presented in this study.

**Scheme 1:**
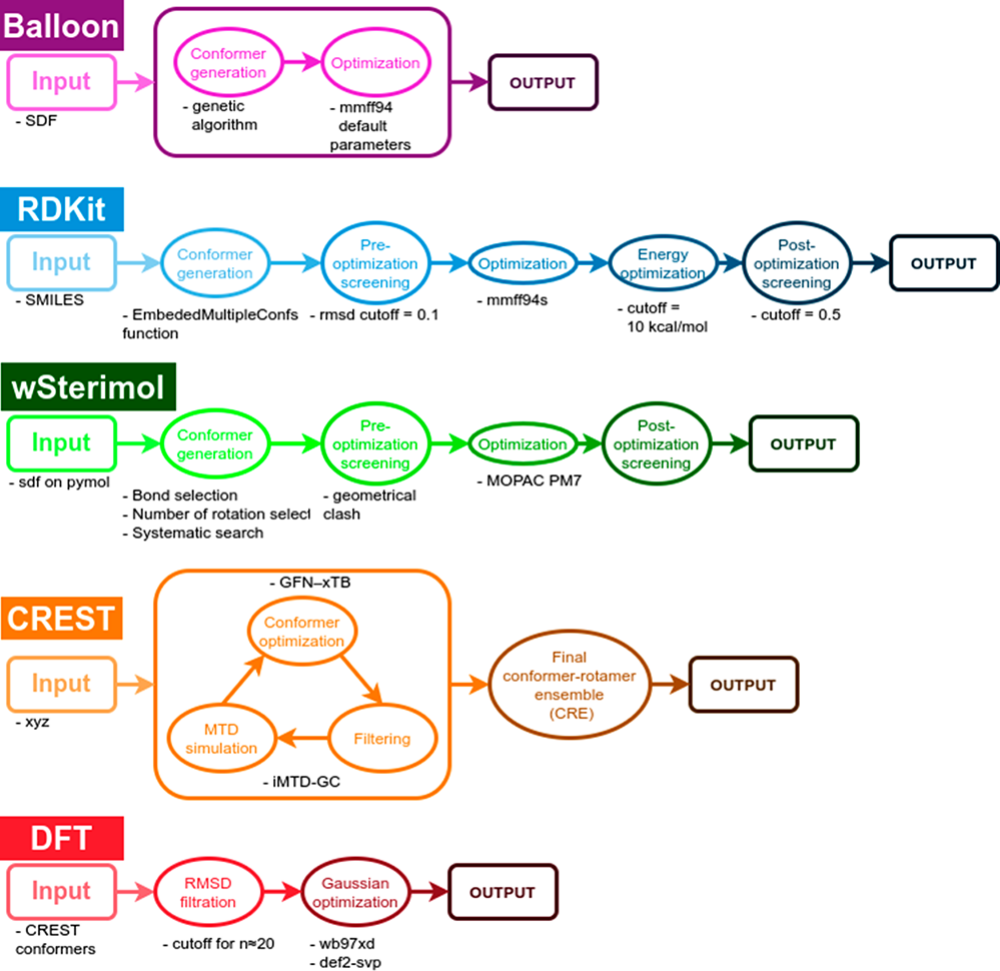
Workflow for the Different Software Used in This Study

A huge number of conformer generators based on different algorithmic approaches are available nowadays. However, for this particular study, only the most used conformational analysis approaches have been chosen to characterize the different Cinchona alkaloid-based PTCs (Scheme 1).

## Computational Details

All the catalyst structures under study were generated with the corresponding SMILES code and preoptimized using RDKit^29^ using the MMFF94 force field so that all the tested programs can use the same preoptimized structures as an input.

For the conformational analysis using Balloon,^30^ the default parameters of the genetic algorithm (GA) and the optimization force field (FF), MMFF94, were used, using the previously optimized structure as an input (SDF format). The default cutoffs were used for the filtration process. All calculations executed with Balloon were performed using the 1.8.0, Apr 10 2021, version of the package.

The conformational analysis performed by using the RDKit required the *EmbededMultipleConfs* function to generate the conformers. The number of conformers was set to be the cube of the number of rotational bonds (nr^3^), the preoptimization RMSD filtration threshold was 0.1 Å, and both options *useExpTorsionAnglePrefs* and *useBasicKnowledge* were activated. The generated conformers were optimized by using the MMFF94s FF, a variation of the MMFF94 FF available on the RDKit that performs better than the default version when planar N hybridizations are present in the molecules. A post-optimization filtration process was performed by means of energy cutoff (*E*_cutoff_ = 10 kcal/mol for unconstrained calculations and 20 kcal/mol for constrained calculations) and root-mean-square deviation (RMSD) cutoff (RMSD_cutoff_ = 0.5 Å). All calculations executed with RDKit were performed using the 2021.03.1 version of the package.

For performing the conformational analysis using wSterimol^31,32^ the molecules were input using PyMOL^33^ (SDF format). The rotating bonds are selected by hand, enabling the rotation of all the nonrigid single bonds in the catalysts, excluding only the bonds forming the quinuclidine substructure, and the number of rotations per bond was set to 3 (ANGLE_COUNT = 3). The software performs a systematic search and optimizes all the generated conformers. The optimization was performed using the MOPAC software^34^ and PM7 FF was selected. The conformers were filtered after being optimized based on their relative energy and geometry similarity. The selected parameters for the optimization and filtration were RMSD_CLUSTER_OPT = 0.5 Å, ATOMIC_MODEL = bondi, RJCT = 0.5, TEMPERATURE = 298, ENERGYWINDOW_CUTOFF = 6.0 kcal/mol, and PRINT_CUTOFF = 6.0 kcal/mol.

The calculations performed using CREST,^35−37^ a utility of the xtb^38^ program, employed the iMTD-GC searching algorithm combined with the default semiempirical quantum mechanical method GFN-xTB, GFN2-xTB, to generate and optimize all the conformers for the input molecule. The energy limit selected was 6.0 kcal/mol, the RMSD cutoff was 0.125 Å, and the energy threshold between the conformer pairs was 0.05 kcal/mol. All calculations executed with xtb were performed using the 6.2 release version of the program.

CREST conformers were filtered using RMSD similarity to obtain a more reasonable number of structures to subsequently calculate at the density functional theory (DFT) level. For this filtration, it has been required first to calculate the RMSD for every possible pair of conformers and second to select a threshold that leaves a certain number of conformers as different as they can be from one another. The remaining structures were optimized at the wb97xd^39^/def2svp^40^ computational level. Harmonic vibrational frequencies were computed at the same level used for the geometry optimizations to confirm that the stationary points were local minima. Calculations were performed using the Gaussian 16 software.^41^

The atomic coordinates for all the generated conformers are provided in Table S1 (unconstrained) and Table S2 (constrained). The evaluation criteria used to assess the different programs under study have been defined as follows.
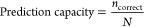
where *n*_correct_ is the number of catalysts in which the predicted most stable conformer is the same as the most stable conformer using the reference method (DFT) and *N* is the number of studied catalysts (18).
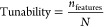
where *n*_features_ is the number of features that can be tuned in each software and *N* is the total number of features that can be modified among all the studied software (conformer optimization method, conformer generation method, cutoff modifications, molecule charge, molecular constraints, solvent selection, and temperature selection).
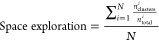
where *n*_clusters_ is the number of clusters in which each program participates for a certain catalyst *in*_total_ is the total number of clusters generated by all programs for a certain catalyst, and *N* is the number of studied catalysts.
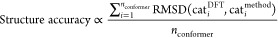
where , *x*_method_ are the coordinates of the generated conformer optimized by the corresponding method and *x*_DFT_ are the coordinates of the generated conformer optimized using the reference method (DFT), *n* = number of atoms considered, and RMSD = 0 corresponds to a perfect grade (10) and the biggest RMSD value (4.70 Å) among all the RMSD calculations performed (12063) corresponds to the worst grade (0). Due to the incredibly large amount of demanding DFT calculations needed, this criterion was calculated for over 10 conformers of 1 catalyst (ph) of each family (OH, squa, and thio).
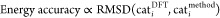
where , where *x*_method_ are the energies of the generated conformer calculated by the corresponding method and *x*_DFT_ are the energies of the generated conformer calculated using the reference method (DFT), *n* = number of atoms considered, and RMSD = 0 corresponds to a perfect grade (10) and the biggest RMSD value (17.31 kcal/mol) among all the energy calculations performed (120) corresponds to the worst grade (0). Due to the large amount of demanding DFT calculations needed, this criterion was calculated for over 10 conformers of one catalyst (ph) of each family (OH, squa, and thio).

The clusterization process has been performed by using the Uniform Manifold Approximation and Projection (UMAP) library for dimension reduction, implemented in Python. This library was combined with the HDBSCAN algorithm to separate the different clusters once the dimension was reduced until a 2D data set has been obtained. For the dimension reduction process, the parameters “n_neighbours” and “min_distance” were tweaked manually for each set of conformers to obtain the best results, “n_components” was set to 2 all the time, and “randome_state” was set to 42. For the HDBSCAN algorithm, the “min_samples” and “min_cluster_size” variables have also been tweaked to obtain the best possible results, minimizing the outliers. The values for these variables are summarized in Tables S3 and S4.

**Scheme 2:**
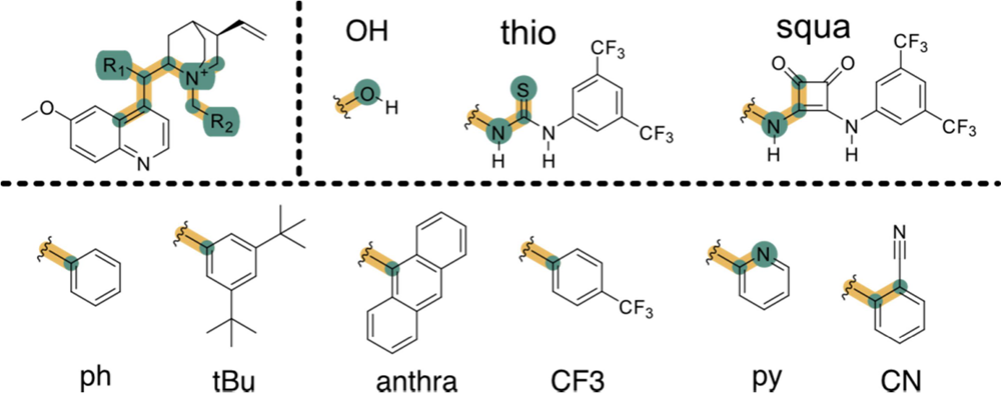
Representative Atoms and Bonds of the Scaffold of the Catalyst and the Different Substituents for the Clusterization Process

This clusterization process has been performed by taking into account the most representative atomic coordinates and dihedral angles of the scaffold of the catalyst and the different substituents (Scheme 2) in order to avoid local symmetry problems and longer computational times. The atoms selected to preserve the direction and orientation of the groups ensure that the different conformers are distinguishable by only using those particular atoms. The data used for the process are the dihedral angles between the bonds that involve the selected atoms and the RMSD calculation between each pair of different conformers using only those atoms.

## Results and Discussion

### Unconstrained Conformational Analysis

We began by conducting the unconstrained conformational analysis of all the different catalysts under study (18 in total) by applying the four different software programs chosen. DFT calculations were carried out for the main conformers obtained from CREST and were added to the global discussion for comparison purposes.

The number of generated conformers of the different catalysts for all the methods under study is summarized in Table 1. As a broad trend, it can be seen that two software with completely different generation methods CREST (metadynamics) and wSterimol (systematic) provide the highest number of conformers, while Balloon and RDKit present the lowest number of conformers for all the catalysts under study.

**Table 1:** Number of Conformers for Each Catalyst Studied with All the Different Conformational Software

complex	Balloon	RDKit	wSterimol	CREST	DFT
cat_OH_ph	47	20	25	164	21
cat_OH_tBu	97	54	29	495	22
cat_OH_anthra	57	30	24	156	21
cat_OH_CF3	57	31	48	246	17
cat_OH_py	58	24	45	144	16
cat_OH_CN	63	43	37	185	17
cat_thio_ph	82	89	272	336	17
cat_thio_tBu	77	114	250	500	19
cat_thio_anthra	61	105	136	430	17
cat_thio_CF3	54	101	218	280	16
cat_thio_py	62	84	334	131	17
cat_thio_CN	40	92	162	432	17
cat_squa_ph	66	121	219	105	15
cat_squa_tBu	38	157	194	258	18
cat_squa_anthra	26	110	95	209	17
cat_squa_CF3	59	163	189	171	15
cat_squa_py	48	28	192	264	18
cat_squa_CN	78	143	477	253	19

If a closer inspection regarding the three different scaffolds (**R_1_** and **R_2_**) of the catalysts under study is made, no general trend could be found (**R_1_** = **OH, thio**, and **squa**). Respecting the substituents, **R_2_ = t-Bu** exhibits the largest number of conformers among the different substituents for the three different types of PTCs. These results are compatible with the fact that the **t-Bu** group displays a high flexibility degree.

**Figure 3:**
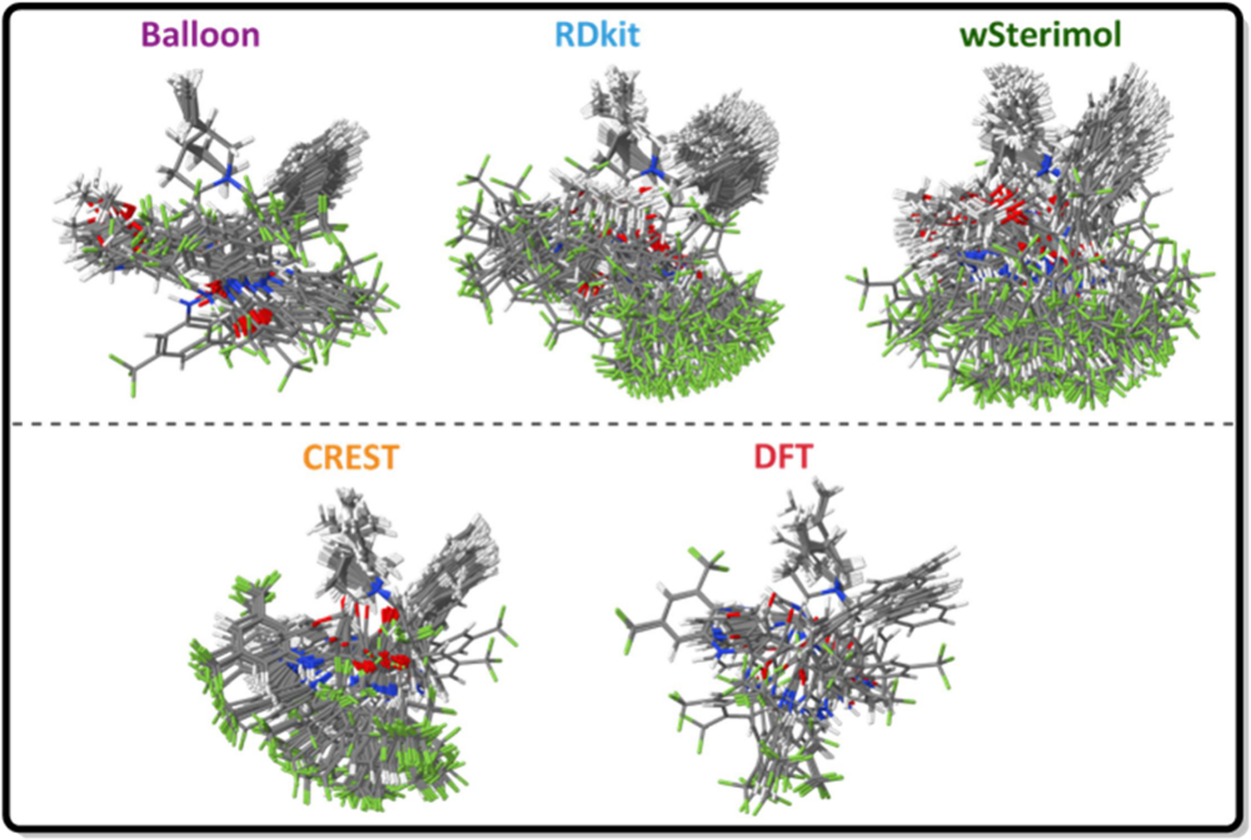
Conformational space explored by all methods under study for cat_squa_anthra.

If the total conformational space explored by the different methodologies under study for a particular case, cat_squa_anthra, is plotted (Figure 3), it is clear that wSterimol and CREST provide a more exhaustive (and possibly more redundant) search. In the case of wSterimol, it rotates all the bonds that can be twisted a certain number of times and then the most interesting ones were selected manually. However, in the case of the CREST software, conformers are generated by extensive metadynamic sampling.

In order to analyze the efficiency of the different software under study and considering the huge amount of data generated, a cluster analysis was carried out using UMAP for clustering the conformers generated by all the programs for each catalyst combined with HDBSCAN for visualization purposes. Ideally, the methodology used should be able to explore the whole conformational space and generate as many clusters (different conformers) as possible, very different from one another.

**Figure 4:**
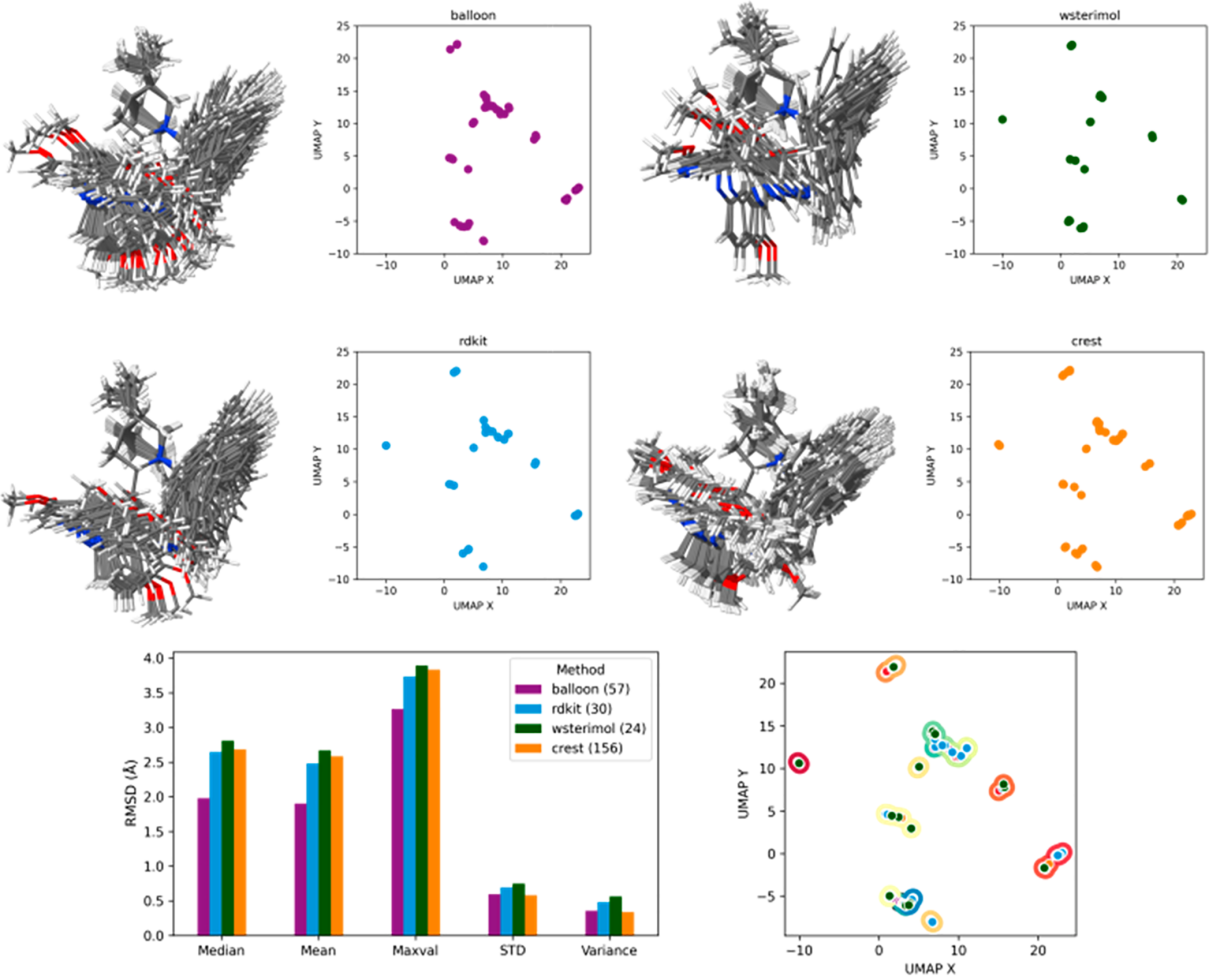
Conformational space explored for cat_OH_anthra.

To obtain a global picture, the number of conformers, the clusters’ information obtained, and all the different statistical data are displayed in Figure 4.

From Figure 4, it is clear that a very similar conformational space has been explored by most of the programs. A similar distribution of points appears when each program’s results are scattered individually (Figure 4 up). Analyzing each individual plot in detail and comparing them with the statistical values, Balloon, with 57 generated conformers, explores only the conformational space corresponding to the right part of the reduced dimension UMAP plots. It is discernible from the Balloon plot that the majority of the points correspond to two big groups of conformers. This uneven distribution and lack of exploration explain the low statistical values that Balloon present among all the different statistical measurements.

On the other hand, RDKit and wSterimol with about half conformers (30 and 24, respectively) explore the same space as Balloon and also explore the extreme left region that did not appear previously. This smaller number of conformers and wider exploration of the conformational space are translated into higher statistical values for all the measurements, as it is highlighted in the graph. RDKit shows a more uneven distribution of the generated conformers, presenting a large concentration in the middle area, explaining the smaller statistics compared with wSterimol. It is reasonable to conclude that wSterimol is the one exploring best the conformational space and obtaining the biggest statistical values because of the systematic search implying the rotation of all the flexible bonds.

Finally, CREST performs the most exhaustive exploration of the conformational space and generates the biggest number of conformers (156). Despite generating so many conformers, they are distributed evenly over all the conformational space and, as a result, the statistical values obtained from such a varied set of conformers are still relatively high.

**Figure 5:**
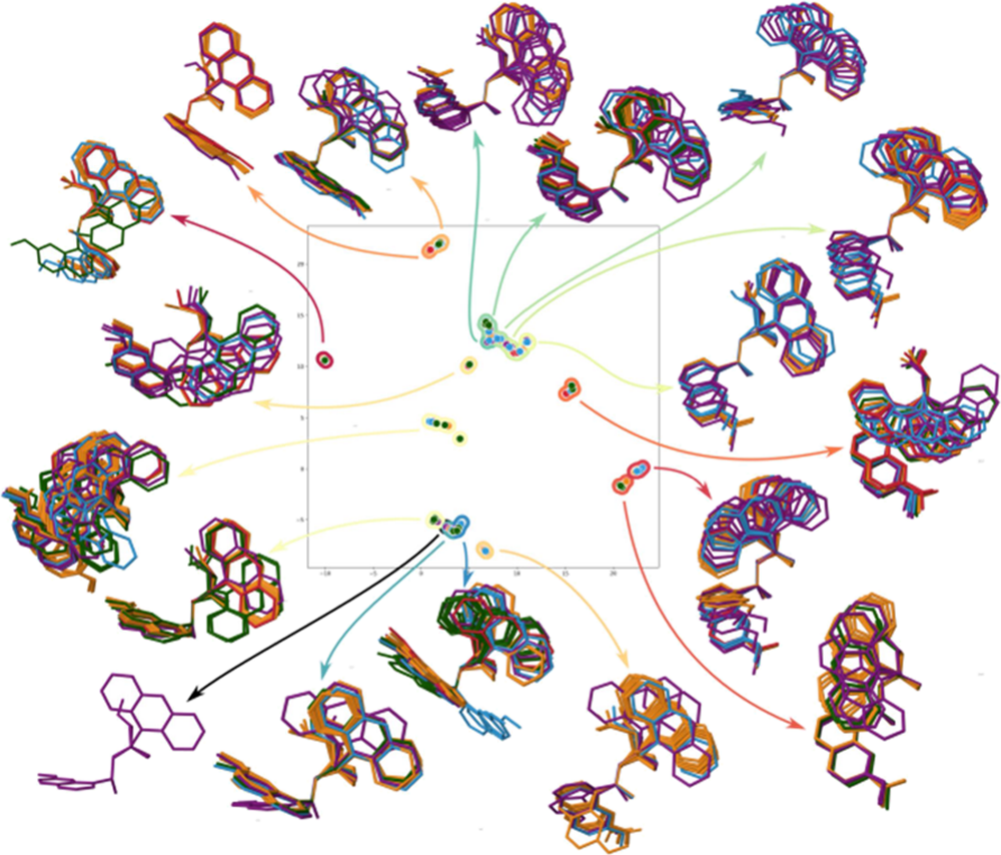
Different conformers corresponding to every cluster generated by Balloon software for cat_OH_anthra.

To illustrate the output from the clustering analysis, cat_OH_anthra has been chosen as a significant example, but the rest of the catalysts are featured in the Supporting Information (Figures S1–S4 and Tables S5 and S6). If a deeper analysis of the different conformers corresponding to every cluster is carried out (Figure 5), a clearer picture is obtained. As a general trend, the conformers are very well collected within each cluster. Since the identification of clusters is a complex process, performed by means of HDBSCAN, some clusters would not be the same that we, as humans, would recognize and select. It can also happen that what could be only one cluster appears as two different ones as it happens with the two greenish-blue clusters on top of the scheme. It was also found that outlier conformers appear as a result of the analysis (black cluster). Despite these problems that can emerge with this intricate analysis, HDBSCAN is, in general, a relatively trustworthy method for separating all the conformers in individual clusters.

**Figure 6:**
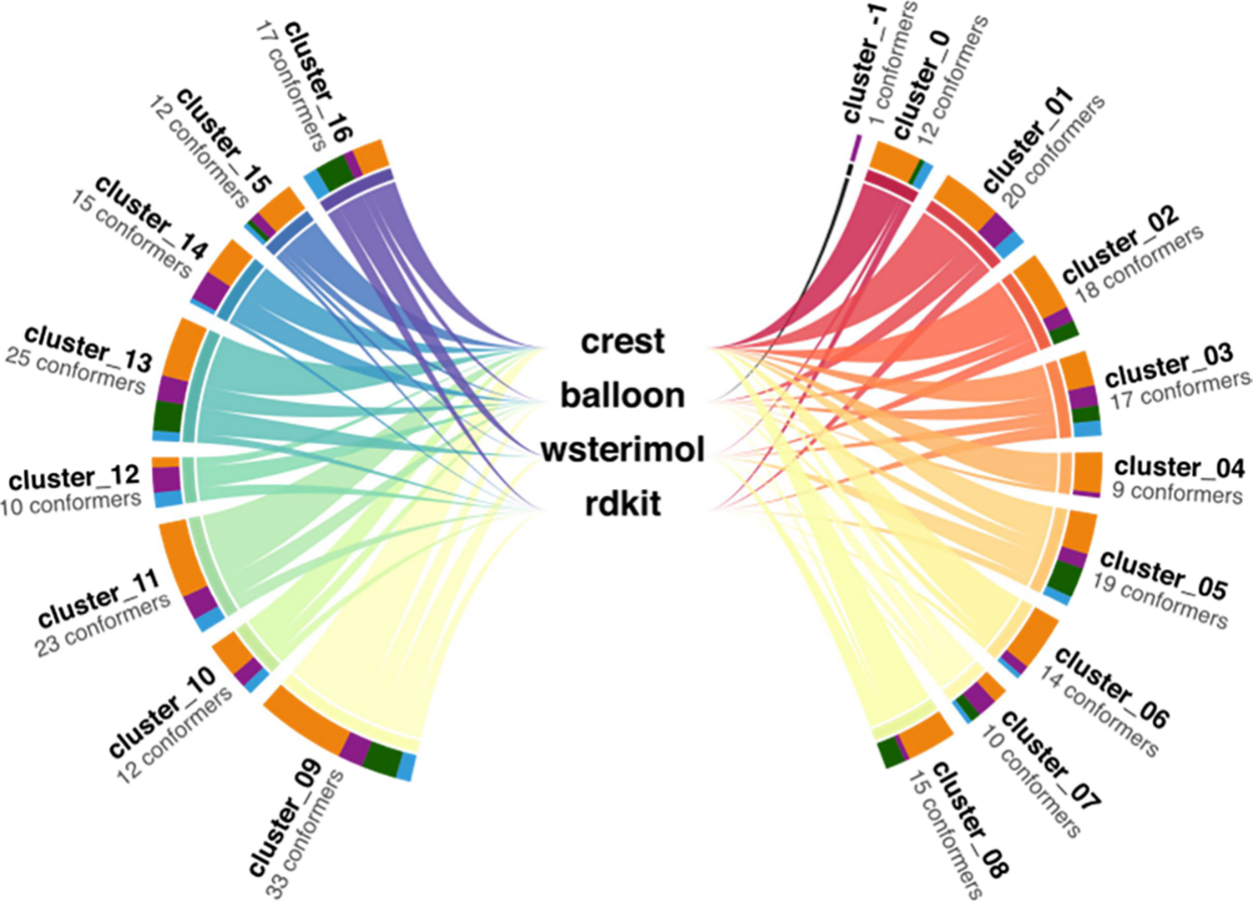
Diagram of the different clusters obtained for cat_OH_anthra and the individual software contribution to each of them.

This cluster information is vital for the evaluation of the different software since it provides the information needed to calculate the space exploration criteria. Figure 6 (and Figure S5) shows, in a very graphical way, how the generated conformers are distributed within the different clusters, how many conformers form each cluster, and the proportion of conformers from each cluster generated by each program.

From the analysis of all the clusters generated for the particular case, cat_OH_anthra, 17 clusters and 1 outlier conformer are obtained. The biggest of those clusters is formed by 33 conformers (cluster_09) and the smaller is formed by 9 conformers (cluster_04). While cluster_09 presents conformers coming from all the studied programs, only Balloon and CREST contribute to cluster_04. As it is shown in the computational details, the number of clusters in which each program participates with respect to the total number of clusters determines its performance when exploring the conformational space. By analyzing this particular case, we can observe that Balloon participates in 16 of the 17 total clusters found for this system. RDKit explored 14, wSterimol 10 of them, and CREST 17, respectively.

Another aspect of the software to be analyzed is its accuracy in optimizing the generated conformers in terms of structural and energy accuracy. Due to the huge number of generated conformers (thousands in total), it is not feasible to optimize all of them at the DFT level and compare the different structures. Therefore, three catalysts were selected (cat_OH_ph, cat_squa_ph, and cat_thio_ph) and 10 different conformers were selected by means of RMSD analysis, confirming that the studied conformers were as different as possible, and reoptimized at the DFT level.

**Figure 7:**
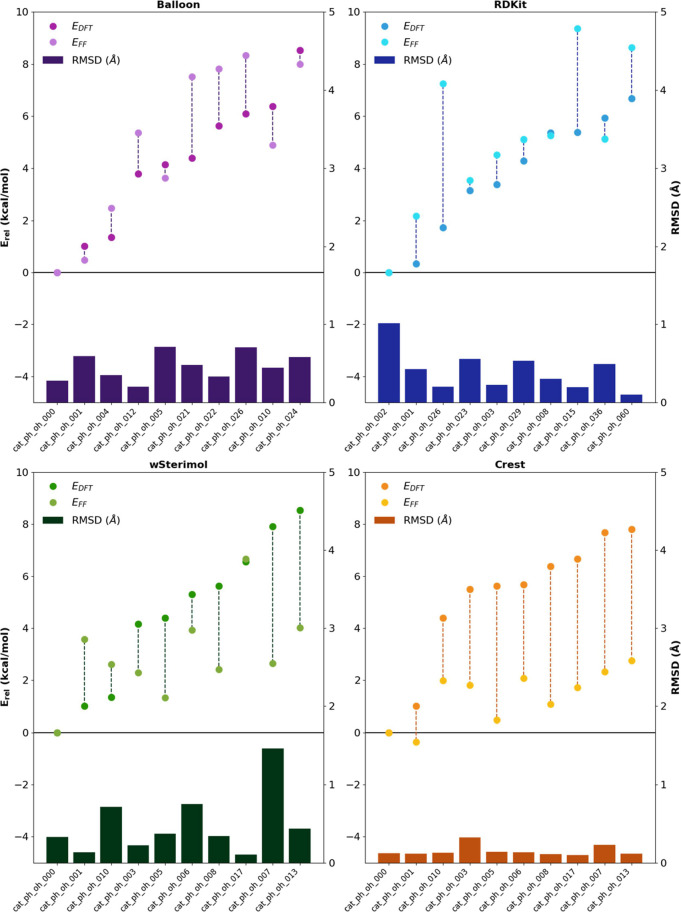
Energy and RMSD comparisons between the studied conformers optimized with different software with respect to the DFT-optimized structures.

The relative energy predictions shown in Figures 7 and S6 can be considered substandard. Balloon and RDKit succeed on average but present significant differences in some particular cases. Those big variations manifest that MMFF94 and MMFF94s FFs present some inconsistencies with some particular interactions exhibited in those conformers.

Despite those general inconsistencies in values, CREST is more consistent in the error and the energy growing trend. For instance, when analyzing the results, CREST presents the best results (7.3) followed by wSterimol (6.5), RDKit (5.4), and Balloon (4.5). This also manifests that the modified MMFF94s FF from RDKit performs better for this kind of catalysts than the default version used in Balloon. It is worth mentioning that CREST, in general, overestimates or favors conformations where π–π interactions are established, over the rest of the conformers, despite getting the best results in terms of energy accuracy.

In terms of structural accuracy, CREST performs exceptionally nicely with the OH substituent, showing extremely small RMSD values. CREST also displays the most consistent results, predicting all the structures with similar accuracy and presenting the smallest differences between the best and the worst prediction.

On analyzing the three catalysts numerically over all the studied conformers, the results were adequate overall, with CREST being the most accurate one when optimizing the structure of each conformer (8.6) followed closely by RDKit (8.2), Balloon (7.7), and wSterimol (7.7). Generally, the CREST optimization method is in very good agreement with DFT-optimized systems.

**Figure 8:**
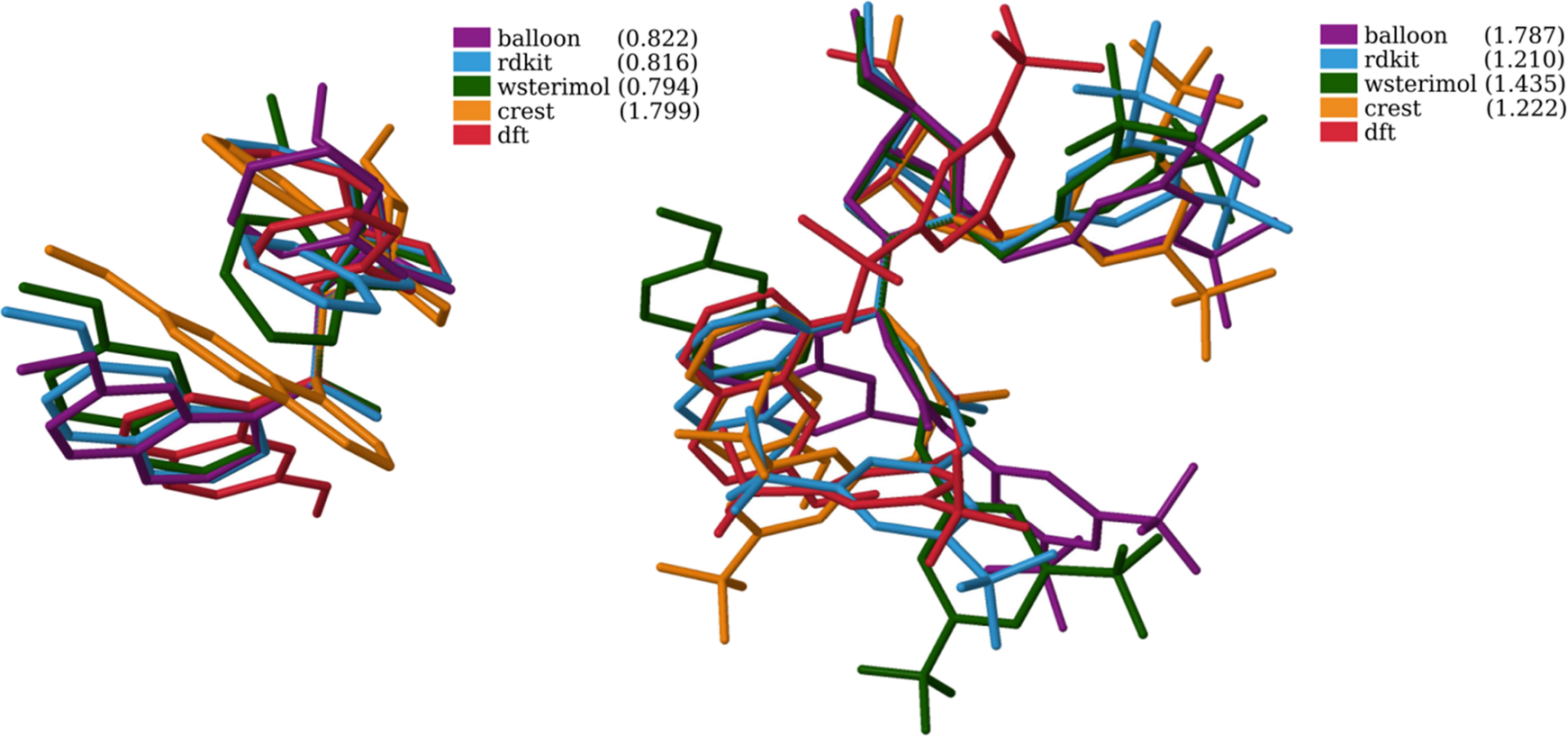
Most stable conformations for cat_OH_py (left) and cat_thio_tBu (right) for each method. Shown in parenthesis are the RMSD values of all the structures with respect to the DFT one, using the same representative atoms than in the clusterization process.

By looking at the most stable conformation obtained from each method (Figure S7), it can be concluded that, as a general trend, the disagreement among the different software and DFT optimizations is, unfortunately, clear and critical, as it can be seen in example, cat_thio_tBu (Figure 8, right). However, for R_1_ = OH, since the scaffold is simpler, a better approximation was obtained, and the most stable conformers present a more similar conformation to the one obtained utilizing DFT (cat_OH_py, Figure 8, left). From Figure 8 (CREST in orange, left), it is worth mentioning that CREST, in general, overestimates or favors conformations where interactions are established, over the rest of the conformers.

To evaluate this disagreement, the prediction capacity criteria were analyzed, accounting for how well each software program predicts the most stable conformation of each of the 18 catalysts studied. The prediction capacity was calculated by comparing the most stable conformation of each individual catalyst, generated with each method, with the most stable reference conformation.

By examining Table 2, it is clear that some particular structures are much easier to predict than others. Those catalysts with R_1_ = OH are, in general, more effortless in predicting the most stable conformer. It is something expected since the OH group is much smaller than squa and thio groups and is not involved in many interactions. On the other hand, catalysts with squa and thio groups are much more flexible and establish a myriad of attractive noncovalent interactions that make these predictions more intricate. In fact, the cat_OH conformational analysis performed was able to successfully predict the most stable conformer in 15 occasions, while only 2 cat_squa and 7 cat_thi were appropriately anticipated.

**Table 2:**
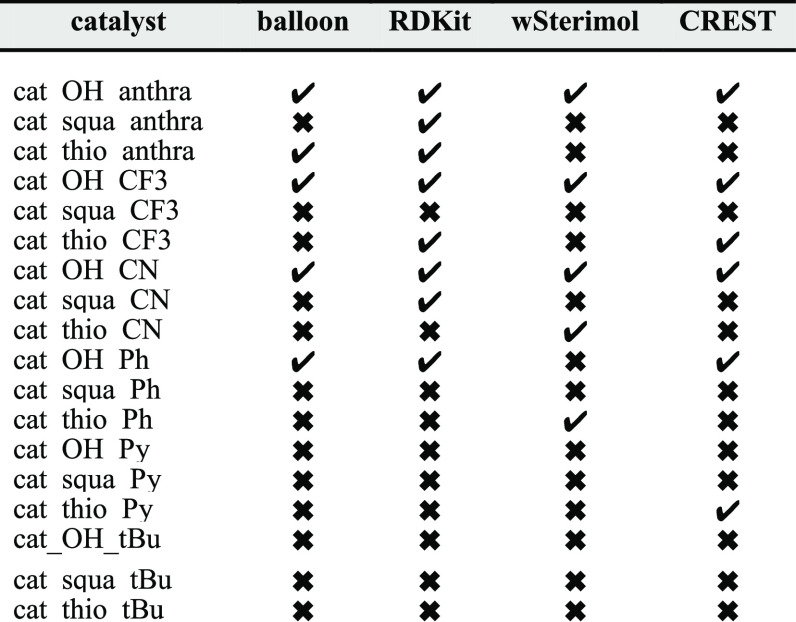
Results on Prediction of the Most Stable Conformation of a Catalyst by Each Studied Software

Focusing on R_2_, the results are also very uneven among all the different combinations, with Ph, Py, and tBu being the substituents presenting less accurate predictions among all the studied catalysts. This can be explained, again, by the fact that those catalysts establish attractive noncovalent interactions that are not well described in the FF and semiempirical methods that the different software use, while in anthra and CF3- and CN-substituted catalysts, sterical issues are much more important in the most stable conformation.

After evaluating these results, it was obtained that RDKit, using the MMFF94s FF, is the software that predicts the biggest number of most stable structures correctly (8) followed by CREST (6), Balloon (5), and wSterimol (5).

In addition, the conformational analysis of a molecule can be affected by external conditions, the method used for generating the conformers, and the optimization method. The conformational analysis software should be as flexible as possible to be able to study systems under different conditions. In order to evaluate the tunability of each software program, a list of requisites was established (see Computational Details section), and each program was evaluated to account for how many of these features can be modified. The result of this analysis (Table S7) concluded that CREST is the most flexible software among all the programs under study (10) and the only one that can modify all the studied features.

**Figure 9:**
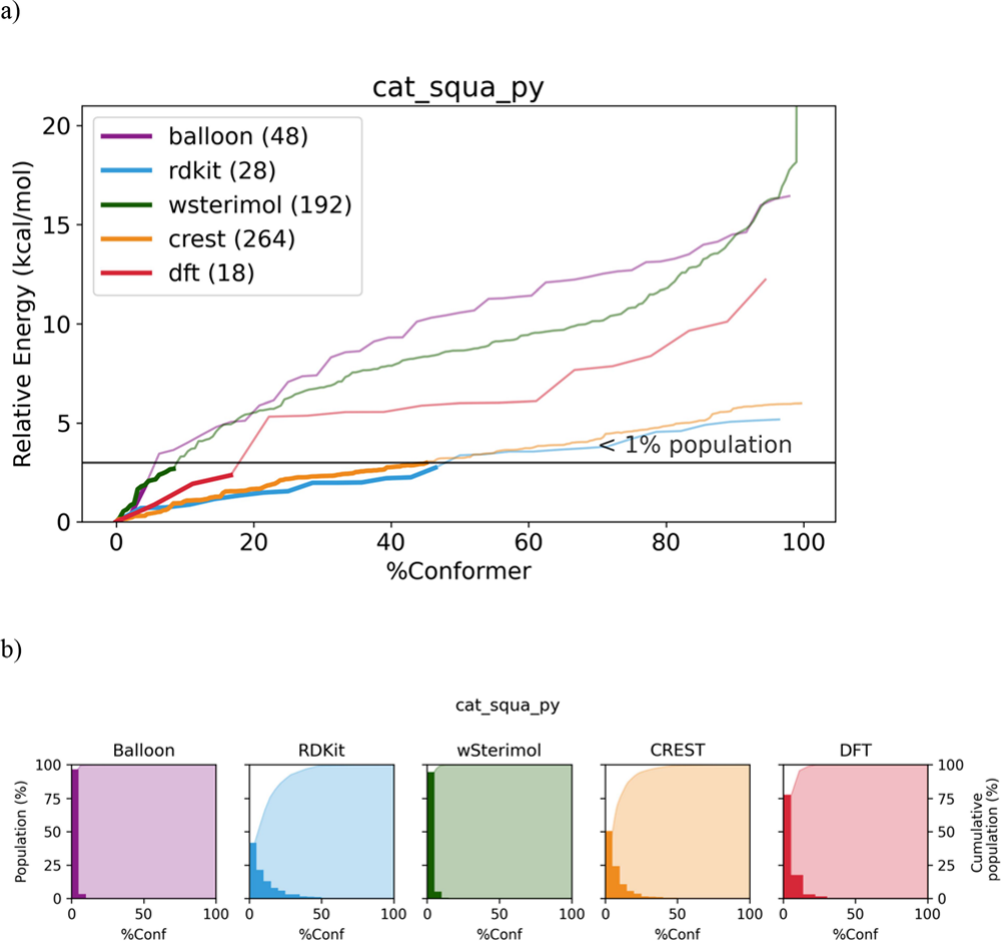
(a) Relative energy of the generated conformers for cat_squa_py with all the studied software. The thick lines represent those conformers that accumulate more than 99% of the Boltzmann population. (b) Shown in bars is the population of the generated conformers with respect to the %Conformers grouped in 5% intervals. The area below the curve, cumulative population of the conformers, is generated with respect to the %Conformers.

It is important to highlight that if a nonconstrained conformational analysis is made with all the different software programs chosen for this benchmark, overall, the *anti–syn* conformation is predominant for all thiourea and squaramide based-catalysts. Therefore, the outcome reached by a regular conformational analysis is not the desired one since only the *anti–anti* conformation is involved in the organocatalytic process. To finish the analysis of the different software under study, a statistical weight of the different conformations in terms of Boltzmann distribution was determined. This analysis explores how many of the generated conformers are statistically relevant and their Boltzmann distribution. The relative energy of the different conformers of cat_squa_py and their Boltzmann population are shown in Figure 9.

This particular case, cat_squa_py, reflects the two extreme different scenarios that could occur when performing a conformational study. As it is shown in Figure 9, conformers generated by Balloon and wSterimol grow extremely fast at the beginning and, as a consequence, less than 10% of the generated conformers represent more than 99% of the Boltzmann population (under 3 kcal/mol). However, RDKit and CREST exhibit a much slower growth and need almost 50% of the total conformers to reach the same cumulative population. In addition, it is clear from the graphics vide supra that both Balloon and wSterimol reach higher energy values, and therefore, could be very appropriate if the aim of the conformational analysis under study is exploring conformations that appear only on that range of energies.

This analysis has been performed for all the catalysts under study (Figures S8 and S9), which lay between these two extreme cases. Overall, the majority of the explored conformers are not statistically relevant, being more pronounced in the case of Balloon and wSterimol.

### Constrained Conformational Analysis

Since the most desirable conformation for the PTC, in order to establish the different interactions with the reactants, is *anti–anti*, the same conformational analysis has been carried out but constraining that particular position.

**Figure 10:**
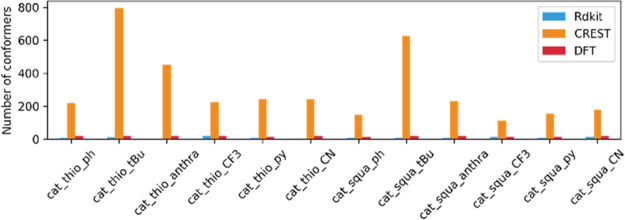
Number of conformers for each catalyst for each software and DFT methodology.

The efficiency of the software under study as well as the same clustering process were performed for the constrained systems. For this analysis, only CREST and RDKit were taken into account since those are the only software in which the position of the catalyst can be constrained. Afterward, a DFT study of the main conformations from CREST was performed for comparison (Figure 10).

**Figure 11:**
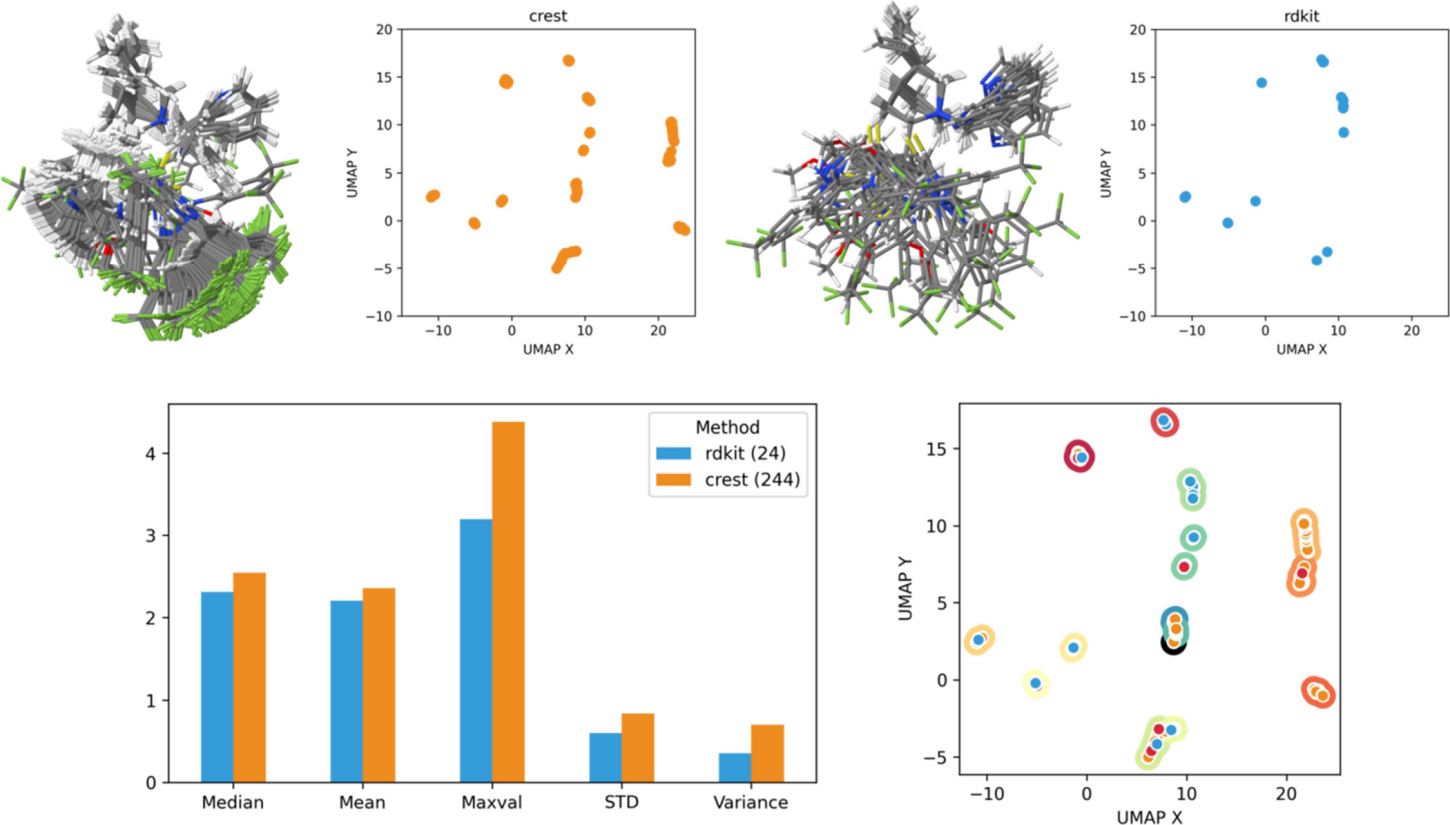
Conformational space explored for cat_thio_py.

To illustrate the output from the analysis, cat_thio_py has been chosen as a significant example (Figure 11), and the rest are depicted in the Supporting Information (Figures S10–S13 and Tables S8 and S9). In order to obtain a global picture, the number of conformers, the cluster information obtained, and all the different statistical data are displayed in Figure 11.

From the clustering analysis, it is clear that RDKit and CREST explore a similar conformational space. At first glance, there is only one area (formed by several clusters and outliers) that RDKit has not explored compared to CREST. This similar exploration has been performed using 10 times fewer conformers in the case of RDKit (24 RDKit and 244 CREST), making it much more efficient in terms of the exploration/number of conformers ratio. Regarding the statistical values, it can be seen that there is a big difference between both methods’ max RMSD value, meaning that CREST has generated a certain pair of conformers much more different than RDKit. This big difference comes from the clusters that RDKit has not explored.

**Figure 12:**
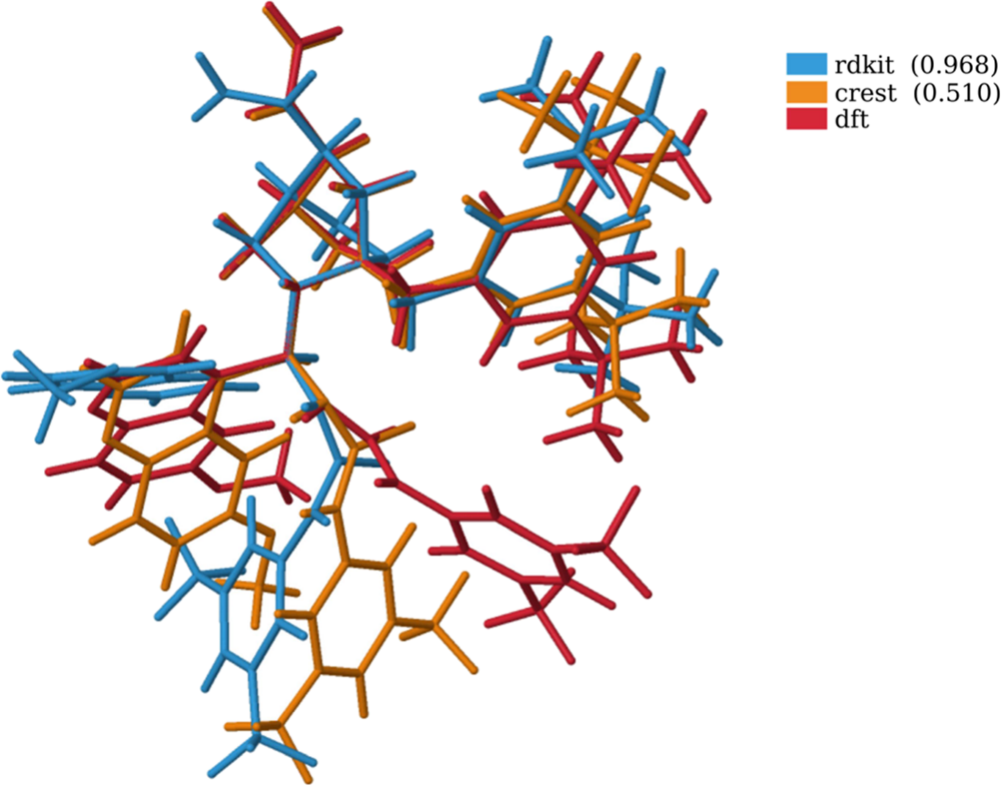
Most stable conformation for cat_thio_tBu. Shown in parenthesis are the RMSD values of all the structures with respect to the DFT one, using the same representative atoms than in the clusterization process.

If a comparison of the most stable structure from RDKit, CREST, and DFT for cat_thio_tBu is made (Figures 12 and S14 for the rest of the cases under study), a better agreement is found. It is clear from the figure that CREST again overestimates the π–π interactions, but in this particular constrained analysis, the main difference among the most stable structures arises from some rotations that can be accessible after DFT optimizations.

**Figure 13:**
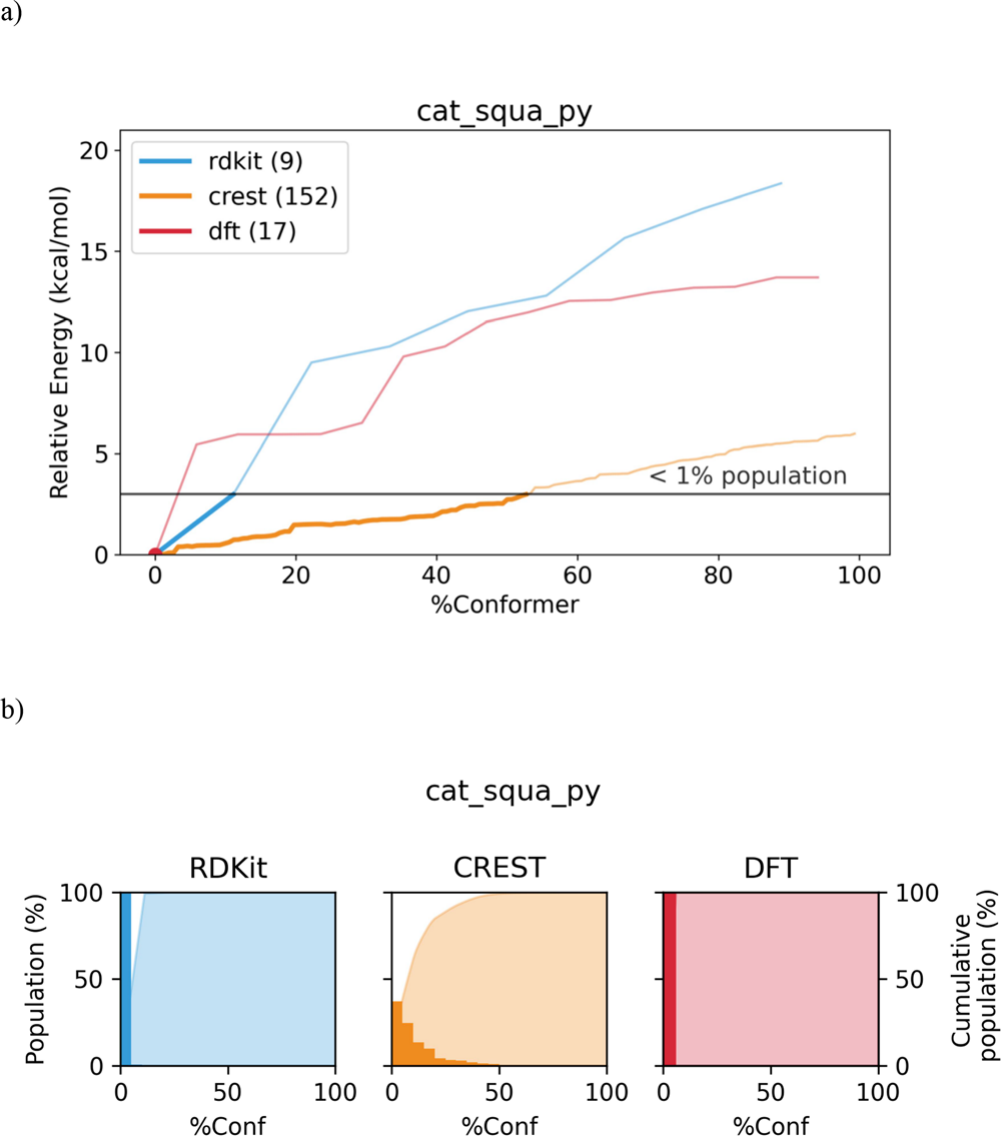
(a) Relative energy of the generated conformers for cat_squa_py with all the studied software. The thick lines represent those conformers that accumulate more than 99% of the Boltzmann population. (b) Shown in bars is the population of the generated conformers with respect to the %Conformers grouped in 5% intervals. The area below the curve, cumulative population of the conformers, is generated with respect to the %Conformers.

If a similar analysis of the statistical weight of the different conformations in terms of Boltzmann distribution was performed for the constrained conformers, a comparable picture was obtained. The relative energy of the different conformers of cat_squa_py and their Boltzmann population are shown in Figure 13.

CREST presents a more gradual growth, needing more than 50% of the total conformers to reach the same cumulative population that RDKit obtains with only 10% of the conformers searched. Overall, the majority of the explored conformers are not statistically relevant, being more pronounced in the case of RDKit.

**Figure 14:**
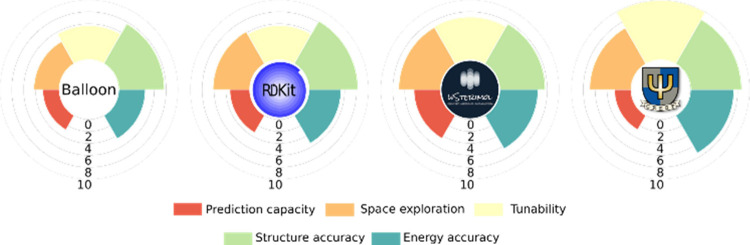
Global grades for each studied software for all the criteria analyzed.

A summary of the grades regarding the different criteria used for each program is depicted in Figure 14. Overall, CREST is the one that obtained the best grades and is the most tunable software, predicting with the best accuracy the structure and the energy of the conformers. However, this particular software is not able to predict the most stable conformer if a comparison is made with the DFT calculation. In addition, CREST usually generates a huge number of conformers in order to explore the conformational space, and for some particular cases, that could not be very efficient. This second problem is very easy to fix by changing the different cutoffs that the program provides.

Finally, Balloon presents the lowest values overall (again, with the default configuration used) but presents a very good agreement when focusing on structural accuracy. The strongest point of this software is its simplicity and the small number of resources that are needed to perform the analysis. This makes Balloon a very good option for generating conformers of a molecule that are going to be used as a starting point for reoptimization at a higher level of theory.

## Conclusions

In this study, a complete exploration of the conformational space of PTCs by means of computational method benchmarking is presented. For this particular study, only the most applied conformational analysis approaches have been chosen to characterize the different Cinchona alkaloid-based PTCs.

This guiding benchmarking study aims to rigorously compare the performance of different conformational methods, determining the strengths of each method and providing recommendations regarding suitable choices of methods for conformational analysis.

When an unconstrained conformational analysis is performed, the most stable conformers correspond to the *anti–syn* conformation for the thiourea and squaramide based-catalysts, providing a nonrealistic scenario for the catalytic process to occur. In addition, if the most stable conformer from every program is compared with the one optimized by means of DFT methods, large disagreements are obtained. Among all the different approaches applied for this benchmarking study, RDKit provides the best balance between efficiency and computing time.

When the constrained scenario is contemplated, only *anti–anti* conformers were studied. RDKit provides again an equitable outcome regarding capability and performance.

Since the main goal of our study is to provide a general knowledge of the different features of a variety of the most common free software for conformational analysis, a general list of pros and cons for each program to facilitate the user the selection of the most adequate software is presented as well as general recommendations. **Balloon**ProsRemarkably
user-friendly (for terminal users). As simple as running
one command in the terminal Multiple possible
modifications (cutoffs, atomic charges, conformer
generation method, symmetry, etc.) Easy to automatize
and include in your scriptsConsInferior
in terms of the outcome obtained Fast energy growth:
a small number of statistically relevant conformers **RDKit**ProsVery versatile
and has numerous options in addition to purely conformational
analysis Easy to automatize
and include in workflows Multiple possible
modifications (cutoffs, atomic charges, conformer
generation method, symmetry, etc.)ConsIt is a library,
not software per se, so it has to be scripted from
scratch Learning curve
more elevated than the rest **wSterimol**ProsSystematic
conformer search ensures that with well-defined
dihedrals, no conformer will be missing Graphical interface.
Good for beginners and users not used to working
with a terminal Energy optimization
can be done directly in DFT (with Gaussian)ConsDifficult
to automatize. It is needed to input the atoms forming
the rotatable dihedrals manually Fast energy growth:
a few numbers of statistically relevant conformers **CREST**ProsEasy to automatize,
works within XTB, and config. files can be reused Many possible
modifications and generation and optimization
options The use of metadynamics
ensures that the conformers are
physically related and are accessible within a selected
energy barrierConsDifficult
to tune the parameters to obtain a reasonable number
of conformers Intermediate
learning curve for specific studies

## General Recommendations

**Balloon**: for a quick and easy qualitative study and to obtain a general idea of the possible conformers of a molecule.
**RDKit**: for including in very automatized workflows (molecule generation, conformer generation, analysis of results, etc.) in Python and reusable codes.
**wSterimol**: for intense systematic studies or studies in which only some specific dihedrals need to be studied.
**CREST**: for exhaustive studies in which the accessibility of the conformers and “physical” meaning are important.

Based on the good results obtained and their capacity for being generalized and automatized, both CREST and RDKit would be the preferred recommendation for general users to include in their daily workflow and research projects.

## Data and Software Availability

All the data and scripts used in the analysis of the results can be found in the Supporting Information: https://doi.org/10.5281/zenodo.7116024
